# Lessons learned from the fifth wave of COVID-19 in Hong Kong in early 2022

**DOI:** 10.1080/22221751.2022.2060137

**Published:** 2022-04-09

**Authors:** Pak-Hin Hinson Cheung, Chi-Ping Chan, Dong-Yan Jin

**Affiliations:** School of Biomedical Sciences, The University of Hong Kong, Pokfulam, Hong Kong

From 31 December 2021 to 23 March 2022, the ongoing fifth wave of COVID-19 in Hong Kong has claimed 6356 lives. The 7-day rolling average of new deaths attributed to COVID-19 per 100,000 people in Hong Kong on 12 March 2022 is 3.73, the highest in the world and much higher than 0.38 and 0.17 in the USA and Singapore, respectively. The numbers of local cases newly reported in the past 24 h by nucleic acid tests and rapid antigen tests (RAT) are 4246 and 7990 on 23 March 2022. The cumulative number of reported cases is 1,075,519 or 14.5% of the population. Taking account of the untested and unreported cases, it is not unreasonable to estimate that up to 30% or 40% of Hong Kong population has already been affected. Generally consistent with this, the daily point prevalence of ongoing SARS-CoV-2 infection in the past 24 days experimentally determined in a real-time community surveillance initiative, in which 10,000 people from 18 districts of Hong Kong are tested by RAT, ranges from 1.25% on 22 March 2022 to 10.76% on 7 March 2022 (https://covid19.sph.hku.hk/dashboard).

Before the fifth wave, Hong Kong had managed to remain free of local cases of COVID-19 for almost 100 days, ascribed to the enforcement of moderate non-pharmaceutical interventions (NPIs) and stringent border control measures, just to be balanced with relatively normal economic activities and social lives. A complete lock down of the city was never an option. Hong Kong had been very successful in containing the Alpha and Delta variants. One of the first cases of human infection with the Omicron variant was reported in Hong Kong in November 2021. The complete sequence of the Omicron variant was quickly determined. The transmission of the virus to another person with no physical contact with the index case through aerosols within a quarantine hotel was also documented [1]. There was no local transmission in the next 40 days until two crew members of Cathay Pacific Airlines brought the virus to our communities. Although we successfully contained the transmission of the Omicron variant from these two sources and that of the Delta variant from hamsters in the pet stores to humans [2], the highly transmissible BA.2 subvariant of Omicron outcompeted our containment efforts. The explosive spreading of BA.2 was retrospectively determined to initiate from an incident in a quarantine hotel, where a South Asian woman was infected by other foreign quarantinees staying in another room and with no face-to-face contact. Airborne transmission was suspected and the incident was thought to occur towards the end of her 21-day quarantine stay. She passed on the BA.2 virus to her husband, who in turn infected in Yat Kwai House, Kwai Chung Estate a cleaning lady, one of the first super-spreaders in the outbreak. The outbreak quickly proceeded to the explosive phase and went out of control under close monitoring. Although multiple NPI measures were enforced before the start of the explosive phase, their effects were in doubt. It is widely believed that NPIs alone, such as social distancing, mass testing for viral RNA, closedown of restaurants and other high-risk places, contract tracing, quarantine and isolation, might be capable of preventing major outbreaks caused by the Alpha or Delta variant, but they might not be sufficient when the Omicron variant is the culprit. The fifth wave is a turning point in the fight against COVID-19 in Hong Kong. Lessons learned from this will be most relevant and beneficial to other regions and countries that stick to a zero COVID-19 policy. Below are six of these hard-learned lessons.

First, the virus and the disease in the fifth wave of COVID-19 in Hong Kong have become very different from those in the early outbreaks in Wuhan or the world. The BA.2 subvariant is the most transmissible but the least pathogenic among all existing strains of SARS-CoV-2. In addition, BA.2 is vaccine evasive and most infections in the fifth wave were breakthrough infections, since 91.3% of the population in Hong Kong had received the first vaccine dose and 81.3% has been injected with the second dose. More than one-third has even been vaccinated with a third dose. In addition, about 500 people were reinfected by BA.2 in the fifth wave. Thus antibodies against SARS-CoV-2 began to rise quickly after breakthrough infection or reinfection and plateaued within 3–5 days after the viral RNA and viral antigen became detectable. This course of antibody production, the course of the disease and the window of virus shedding have all been shortened by at least 2 days when compared to the infection of an immunologically naïve individual with the ancestral strain of SARS-CoV-2. These antibodies are thought to neutralize the virus and further relieve the symptoms if there are any. As a result, more than 99% of the BA.2-infected cases in the fifth wave of COVID-19 in Hong Kong were either asymptomatic or mild. In these people, the disease was at least not more severe than flu or common cold. Due to the protective effect of vaccination, it was much less severe in most cases. At any time point, there have been less than 300 cases requiring mechanical ventilation and the use of Intensive Care Unit (ICU) all over Hong Kong. Many infected individuals are unaware of the infection because they are either asymptomatic or have mild symptoms. This has also made the identification of infected individuals and the execution of the zero COVID-19 policy more difficult. To those who have been fighting with other variants of SARS-CoV-2, it is important to bear in mind the major difference between BA.2 in transmissibility and pathogenicity.

**Figure 1.**
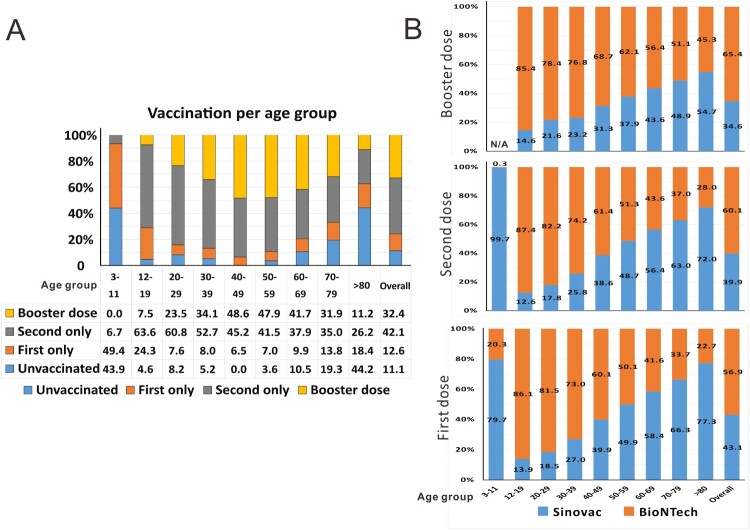
COVID-19 vaccination status in Hong Kong as of 17 March 2022. (A) The percentages of people in the overall population and each age group without or with injection with the first dose only, second dose only or booster dose of COVID-19 vaccine (including both Sinovac and BioNTech) in the period of 26 February 2021 to 17 March 2022. (B) The percentages of Sinovac and BioNTech injected as first, second or booster dose in each age group and in the overall population (Original data: https://www.coronavirus.gov.hk/eng/index.html).

**Figure 2.**
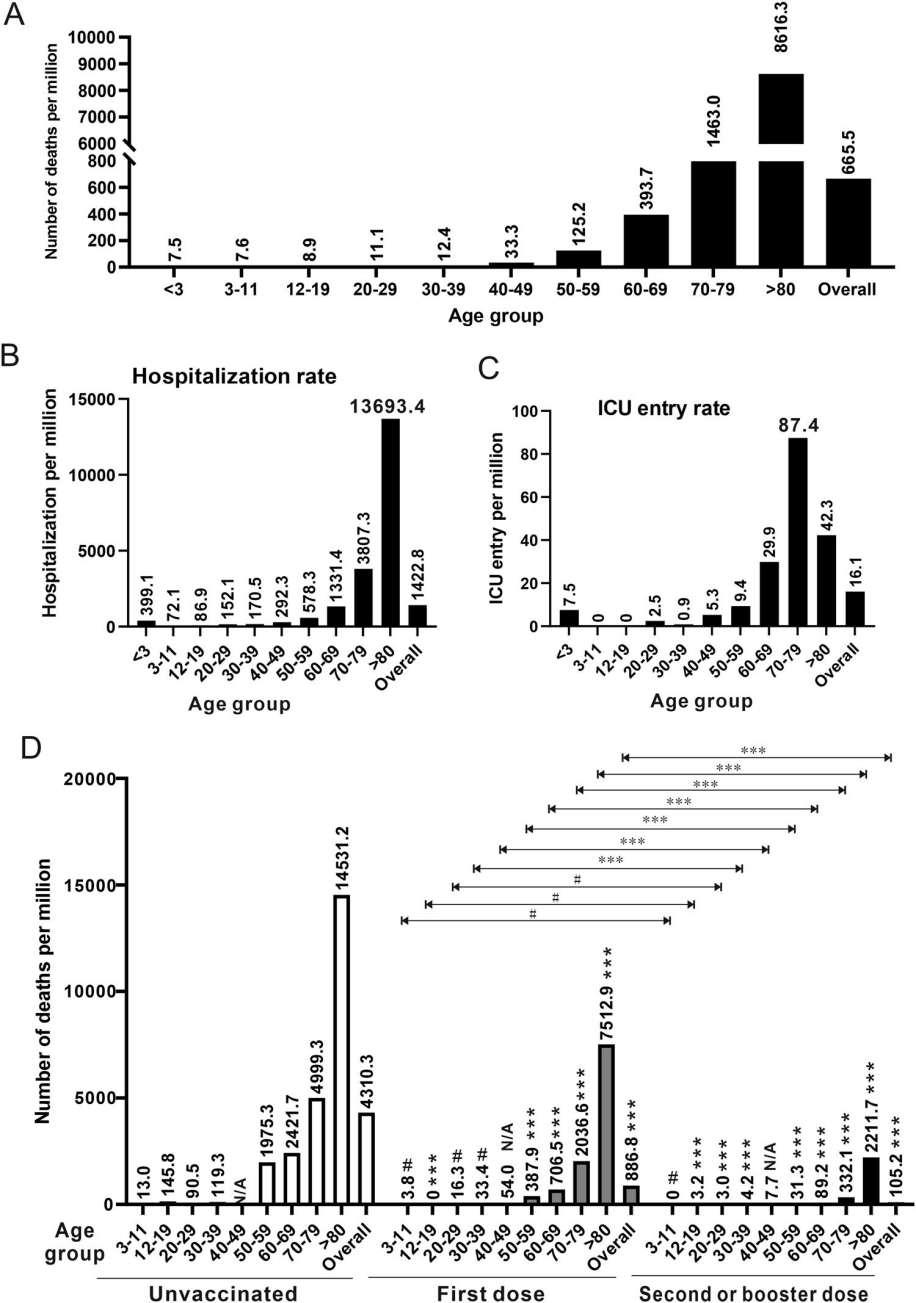
Mortality of COVID-19 during the fifth wave is reduced with vaccination. (A) The overall mortality rate (number of deaths per million as calculated by dividing the number of deaths by the number of people of the specific population) and mortality rates of all age groups in the period of 31 December 2021 to 17 March 2022 in Hong Kong. N/A: not applicable. (B) The hospitalization rate (hospitalization per million) of each age group. (C) The ICU entry rate (ICU entry per million) of each age group. (D) The overall mortality rate (number of deaths per million) and mortality rates of all age groups without or with first dose or second-booster dose of COVID-19 vaccination. Unpaired Student’s *t*-test was performed to judge the statistical significance of the difference between groups. Symbols (***P < 0.001; **P < 0.01; *P < 0.05; #*P* > 0.05.) above bars represent *P* values comparing the vaccinated age group with the unvaccinated counterpart of the same age. *P*-value comparing the same age group having first dose vaccination with second/booster dose vaccination was indicated above horizontal lines. N/A: not applicable (Original data: https://www.coronavirus.gov.hk/ eng/index.html).

Second, the exceedingly high death rate in the fifth wave of COVID-19 in Hong Kong is attributed to the tragic loss of elderly who were unvaccinated and not fully or effectively vaccinated elderly. Although people in the age groups of 60–69, 70–79 and >80 account for only 14.5%, 7.6% and 5.4% of the population in Hong Kong respectively, they contributed to 13.5%, 20.9% and 51.4% of hospitalized cases as well as 8.4%, 16.6% and 70.8% of deaths in this wave. In other words, 85.8% of hospitalized cases and 95.8% of deaths were from the elderly in these three age groups. Approximately 90% of people deceased in this wave did not receive two doses of either BioNTech mRNA vaccine or Sinovac inactivated vaccine. Whereas about 90% of all people in Hong Kong were vaccinated with at least one injection as of 17 March 2022, the percentage of vaccinated elderly of over 80 was 55.8% (Figure 1A). At the beginning of the outbreak in early February, less than 20% of people over 80 were vaccinated. This is in stark contrast to the vaccination rate of more than 60% against seasonal influenza in the same group. The number of vaccinated people was slightly better in the age group of 70–79 but it was also on the low side. In these two age groups, more than 70% and 60%, respectively, of the elderly received the Sinovac vaccine (Figure 1B), which is known to have very weak protection after one injection and to be much less protective against the Omicron variant after two or even three injections [3]. Tragically, the mortality rates (per 1,000,000 people) in these two age groups are 8616.3 and 1463.0, compared to 393.7, 125.2 and 33.3 in the age groups of 60–69, 50–59 and 40–49, respectively (Figure 2A). In keeping with this trend, considerably more people over 60 were hospitalized (Figure 2B) or admitted to the ICU (Figure 2C). If we compared the mortality rates of unvaccinated people over 80 (14531.2 per 1,000,000) with that of the same age group of people who received second or third dose of vaccine (2211.7), there was a 6.6-fold difference. Likewise, a 15.1-, 27.1- or 63.1-fold difference between unvaccinated and fully vaccinated was seen for the age group 70–79, 60–69 or 50–59 (Figure 2D). The benefits of vaccination were significantly greater in the relatively younger groups. It remains to be further analysed how much of this might be attributed to the age and to the choice of vaccine, since increasingly more people were immunized with BioNTech in the younger groups (Figure 1B). Notably, very few of the recipients of three injections of BioNTech or two injections of Sinovac plus one injection of BioNTech had died. In other words, booster injection with BioNTech not only reduced the chance of infection with BA.2 but also protected against severe disease and more importantly death. This is generally consistent with the real-world data in Singapore, where booster injection with mRNA vaccines led to 24.8 fold reduction in mortality in the age group of >80 when compared to the non-fully vaccinated or 4.1 fold reduction when compared to those who received two injections (https://www.moh.gov.sg/covid-19/statistics). Even when breakthrough infection was seen in a relatively small subset of people who were booster vaccinated with BioNTech, they were either asymptomatic or had mild disease. Before the arrival of next-generation Omicron variant-specific vaccines, booster injection with a highly effective vaccine such as BioNTech should be the best way to prevent infection, severe disease or death. Several emergency measures have been implemented to reduce the deaths of elderly, including: (a) emergency vaccination; (b) adopting the close-looped management system of Chinese style in elderly care homes to minimize the contact of the elderly and their caretakers with the outside world; (c) prescribing Paxlovid and molnupiravir to high-risk patients once infected; (d) performing RAT on every elderly on a regular basis so that infected people can be identified and isolated as early as possible; (e) assigning all elderly patients who have to be hospitalized to designated hospitals where they will be taken better care of and (f) reverse quarantine some high-risk elderly who have not been infected. It was felt that immunizing the elderly now might already be too late, particularly with an inactivated vaccine that takes more injections to have weak protective effect against Omicron. Opposite to what has been suggested by some experts, it has been recommended strongly that the BioNTech vaccine should be used in the elderly, since the beneficial effect might be more immediate and pronounced. Elderly people are considered immunocompromised. Regardless of the type of vaccine used, they must be immunized with as many vaccine doses as required until sufficiently high titres of neutralizing antibodies are detected. Plausibly, three or more doses of mRNA and inactivated vaccines might be needed. The underlying causes of vaccine hesitancy in the elderly are manifold and complicated, including complacency and wrong judgement from the elderly and their family members partially due to the previous success in Hong Kong’s effort to contain SARS-CoV-2, lack of strategy and plan from the government to design and implement a vaccination campaign in the elderly, as well as misinformation from multiple sources concerning the efficacies, side effects and long-term impact of SARS-CoV-2 vaccines. Singapore has a comparable total number of confirmed cases of COVID-19 (about 1M as of 23 March 2022), but its total number of deaths is 1220, only 19.2% of the number of Hong Kong. The percentages of people who received two doses of mRNA vaccine or three doses of inactivated vaccine in the age groups of 60–69, 70–79 and >80 in Singapore are 97%, 96% and 94%. In addition, only about 1% of Singaporeans chose inactivated vaccines. Effective vaccination makes the difference. To some extent, the deaths of unvaccinated elderly in Hong Kong are an expected tragedy. With their invaluable lives, the elderly people who died in the fifth wave in Hong Kong had taught the world the hardest lesson about protecting the elderly and the most vulnerable with effective vaccination against SARS-CoV-2.

Third, BA.2 infection in children is common but not particularly more pathogenic as seen in the fifth wave. Since the vaccination program in the pediatric group had just started when this wave broke out (Figure 1A), it was not surprising that children in the age group of 0–19 accounted for about 8% of the whole infected population. The infection rate in children was not higher than those of the other groups. Although the deaths of several BA.2-infected pediatric patients have attracted widespread concern and panic from the general public, BA.2 infection in children was not particularly severe or lethal. There were nine deaths, which is 0.81 per 100,000 in children of 1–19. This was comparable to or lower than the numbers of 7 (0.87), 14 (1.24) and 35 (3.06) in the age groups of 20–29, 30–39 and 40–49. Similar trends were also seen when we compared the mortality (Figure 2A) as well as the hospitalization (Figure 2B) and ICU entry (Figure 2C) rates. In particular, the mortality rates of 7.6 and 8.9 per 1,000,000 in the 3–11 and 12–19 groups were comparable or lower than those of any other group (Figure 2A). Notably, all children who died had underlying conditions. As seen in the age group of 12–19, vaccination with two or three doses was beneficial in reducing deaths by 45.6-fold (Figure 2D). Thus in contrast to the misconception that BA.2 had killed many children or caused more severe diseases in children during the fifth wave in Hong Kong, BA.2 infection in children was mostly mild or asymptomatic as in other parts of the world. Furthermore, vaccination is the most effective way to protect children against SARS-CoV-2 infection, severe COVID-19 disease or death.

Fourth, RAT has played an important role in identifying infected individuals and safeguarding a COVID-free working environment in the fifth wave. High-quality RAT reagents, with close to 100% sensitivity for people with a *C*_t_ value of lower than 25 in RT-qPCR test of viral RNA together with close to 100% specificity, have been available in the market. RAT is particularly useful in settings where the number of infected people is high. Because viral loads usually rise rapidly to high levels in the initial phase of infection, both RT-qPCR and RAT have good performance in picking up infected individuals in this phase. In the convalescent phase, RT-qPCR might pick up more people with a *C*_t_ value of higher than 25. However, most of these people have neutralizing antibodies and are no longer infectious. Since RAT positivity correlates better with virus shedding and infectivity, RAT is more helpful in identifying infected individuals, particularly those who are potential super-spreaders. The collective results of a series of daily RAT tests within 5 days are more sensitive and reliable in early diagnosis of SARS-CoV-2 infection, if compared to single or double RT-qPCR tests of viral RNA conducted in the same period. Sample pooling is one common strategy for the RT-qPCR test of SARS-CoV-2 RNA. The sensitivity might be compromised when 10 or 20 samples are pooled for one test. Sample pooling is not feasible when many people have already been infected as in Hong Kong. RAT is both more sensitive and specific in these settings. Because RAT result is available in 15 or 20 min, rapid diagnosis and isolation of infected subjects can be achieved. At the early phase of the fifth wave, thousands of people were queuing for hours to be tested for viral RNA. This provided ample opportunities for viral transmission. Meanwhile, the turnover time for viral RNA test was much longer due to the exceedingly high demand, ranging from 3 to 7 days. This rendered the test pointless and a waste of resources. As discussed above, infection with BA.2 progresses very rapidly. The recipients of the test had already become much less contagious after 3 days when the viral RNA test result was available. They should have recovered completely and cleared the virus after 5 days. The performance and utility of RAT are much more impressive in these settings. The need for mandatory universal RNA testing has been debated during the fifth wave. It was proposed that three consecutive RNA tests would be performed on everyone within either 3 weeks or 9 days as was recently done in the nearby Chinese city of Shenzhen. This is extremely costly and should be ruled out immediately when people were waiting for days to receive the results of their viral RNA test. It is also pointless when gathering of people should be absolutely avoided, quarantine facilities are short of supply and the communities are already full of infected people. Due to the high number of infected people, it is unlikely to identify all of them with only three rounds of universal viral RNA test or to achieve zero COVID-19 after this. One counterproposal is to have everybody to perform daily RAT for 5 or 10 days. This could be executed more than once with the different goals of reducing infection and eliminating the virus in the community. RAT was finally recognized to be a valuable tool complementary to the viral RNA test in the ninth edition of the Guidelines for Diagnosis and Treatment of COVID-19 in China. We believe that RAT will prove useful in combating the coming outbreaks of COVID-19 in China.

Fifth, the healthcare system in Hong Kong has been under tremendous pressure during the fifth wave but has not collapsed and should not collapse. The tsunami of cases has created a huge burden. More than 15,700 or about 20% of all public hospital staff have been infected, but half of them have returned to work as of 17 March 2022. It has been debated as to whether the healthcare system has collapsed, is on the verge of collapse or has stretched to its limit. Whereas some appealed to the public that we should protect our healthcare system so that it does not collapse, others argued that the healthcare system should be built to protect the people and not the opposite. Actually, these should be interdependent. Our healthcare system can protect the people only when it has not collapsed. As mentioned above, at any single time point since the beginning of the fifth wave, the total number of patients who suffered from severe disease requiring mechanical ventilation and had to stay in the ICU is below 300. The vast majority of patients had mild disease and did not require special medical care. Many were hospitalized just for precaution. Thus, if the healthcare system in Hong Kong collapses in the event of the fifth wave, it must be due to misjudgment, wrong decision and wrong allocation of resources. However, it is just impossible that all those who want to be hospitalized can stay in hospital. This message has to be made clear to every stakeholder. Right before the start of the fifth wave and to satisfy the criteria for mainland China to open the border to Hong Kong, we had used a *C*_t_ value of 40 in viral RNA detection for the release of infected individuals from the hospital as per advice by experts from the other side. People who were tested positive again in viral RNA analysis after they had recovered from the infection were also called back to the hospital. This resulted in the excessive occupation of beds by people with no medical need in the initial stage of the fifth wave. In response to the rapid increase of confirmed cases, we had revised this to use a *C*_t_ value of 33 and then 30 for the release of patients from the hospital. Finally, *C*_t_ values would only be used as a reference and patients could be released completely at the discretion of the attending physician if the release is considered to benefit the patient, people in contact with the patient and the community. An appropriately designed risk stratification and triage system must be in place so that only patients in real need of medical help are hospitalized. This has to be adjusted constantly to meet the need of patients and to make best use of the medical resources including the hospitals. At one point, priorities for hospitalization were given to the elderly (Figure 2B), pregnant women, people with underlying diseases and children. This was later modified to triage these patients further based on their risk factors. It is the risk stratification and triage system that help protect our hospitals so that both COVID-19 and non-COVID-19 patients are taken good care of. Major improvements to this system are required so that it can cope with the ongoing fifth wave and any future waves. In this connection, mobilizing the private sector which has ample resources is an important task at present and in the future. Many non-hospitalized patients with mild symptoms suffered from anxiety or other mental health problems and were also the victims of misinformation. Their medical needs were met in the designated clinics run by the government. A hotline was also set up for counselling and report of red flag signs and symptoms. Ideally, private practitioners, psychologists, psychiatrists, traditional Chinese medicine doctors and social workers should work together to provide better support to these patients. In this regard, virtual medical consultation will be an attractive option. Our fight against the fifth wave will be more successful and our society will be in a stronger position if this big group of infected people is happy and healthy both physically and mentally. Better communication is the key.

Sixth, to stay home safe is a viable option for many people during the fifth wave. The StayHomeSafe scheme implemented is not only cost effective but also achievable for many infected people and their close contacts. With heightened infection control measures, family clustering, as more frequently seen in earlier outbreaks [4], was observed only in one-third to one-half of reported cases. The attack rate within the family was also lower. Because the window of virus shedding in breakthrough infections of BA.2 is shortened, the time required for home isolation or home quarantine should also be shorter. In addition to university dormitories, hotels and public residential buildings, quarantine facilities and makeshift hospitals have been rapidly built for Hong Kong with the kind help from mainland China. However, due to the exceedingly high number of cases and close contacts, the quarantine and isolation facilities would never be sufficient. Home isolation and home quarantine are the only available options. As mentioned above, the whole fifth wave started as a result of one single case of accidental infection in a quarantine hotel. This reminds us of the risk and the utmost importance of ventilation and infection control in quarantine facilities. One option is to reserve the quarantine facilities for those who have a desire or need to use them due to cohousing with high-risk family members or lack of private space at home. On one hand, BA.2 is highly transmissible, and accidents could happen in quarantine facilities. On the other hand, with extreme caution and effective infection control, transmission between family members might be successfully prevented. Even for mandatory quarantine and isolation in public facilities, the duration of stay should not be made unnecessarily long. Shortening the stay at public facilities will make the best use of valuable resources without increasing the risk of accidental infection.

The chance for major outbreaks of Omicron in areas where most people have never been exposed to the variant is relatively high. Better preparation for such outbreaks is necessary. When most people in the world have acquired some immunity against Omicron through natural infection in addition to vaccination of as many people as possible, we will embrace the ending of COVID-19 pandemic. We might still have obstacles and challenges ahead, but the light at the end of the tunnel is already in sight as in many parts of the world. The Hong Kong lessons shared here should be instructive and useful for regions that may anticipate Omicron-based outbreaks of COVID-19.
